# Pathology of Senegalese breast cancers

**DOI:** 10.11604/pamj.2019.34.67.17993

**Published:** 2019-10-02

**Authors:** Megan Burke Fitzpatrick, Mara Hester Rendi, Nancy Barbara Kiviat, Pape Toure, Amadou Dem, Papa Salif Sow, Stephen Edward Hawes, Qinghua Feng, Kimberly Heller Allison

**Affiliations:** 1 Stanford University School of Medicine, Stanford, CA, USA; 2 University of Washington, Seattle, WA, USA; 3 Institut du Cancer, Université Cheik Anta Diop (UCAD), Dakar, Senegal; 4 Service des Maladies Infectieuses Ibrahima, Diop, Mar, CHNU de Fann de Dakar, Sénégal

**Keywords:** Breast cancer, Africa, Senegal

## Abstract

**Introduction:**

Breast cancer is among the most common cancers among women in most of Africa. However, features of histologically confirmed breast cancers presenting in specific regional populations is limited. Our study describes the clinic-pathologic features of invasive breast cancer diagnosed in women undergoing biopsy for a clinically apparent mass in Senegal, West Africa.

**Methods:**

A prospective cohort of 522 Senegalese women presenting consecutively to Dantec Hospital (University of Dakar Tumor Institute) with a breast mass were included in the study cohort. Demographic data was collected by survey and 197 (37.7%) core needle biopsy-confirmed invasive breast cancers available for review were subsequently centrally reviewed at the University of Washington in Seattle to further to characterize the pathologic features and to perform immunohistochemistry for ER/PR and HER2.

**Results:**

Seventy six (76.1%) of the 522 Senegalese women presenting for biopsy of a clinically apparent breast mass were diagnosed with invasive breast cancer. The average age of a woman with invasive cancer was 46 years old, and most (83%) presented with Stage III or IV disease. The predominant histologic subtype among the 197 biopsy-confirmed cancers was invasive ductal carcinoma (98%), with few cases of invasive lobular carcinoma (2%). Cancers were classified into four clinically relevant treatment IHC groups by combined ER/PR status and HER2 status as follows: ER-/PR-, HER2- (n=92; 46.7%), ER-/PR-, HER2+ (n=20; 10.1%), ER+/PR+, HER2- (n=76; 38.6%) and ER+/PR+, HER2+ (n=9; 4.6%). Age at time of diagnosis was similar between these four subgroups although more HER2 positive cases were pre-menopausal (p=0.05). Stage of disease at presentation differed by IHC group (p=0.008), with HER2+ cancers significantly more likely to present with stage IV disease than other IHC groups, including ER-/PR-, HER2-. There were no significant differences between groups by age group, ethnicity, place of residence or birth, or parity.

**Conclusion:**

Our analysis of breast cancer cases in Senegal shows a distribution of clinically relevant IHC groups like that seen in the few prior studies of breast cancer in West Africa, with higher frequencies of triple negative cancers than in most United States and European populations. Mean age at presentation, delayed presentation, and genetic/regional risk factors likely influence these differences. A better understanding of the frequencies of the pathologic features of breast cancers in the West African population may help guide future genetic studies as well as appropriate clinical management of breast cancer in these populations.

## Introduction

Breast cancer is the most common cancer affecting women in Africa (100per 100,000), and is the second leading cause of cancer deaths (49 per 100,000) [1]. Western African countries have an average estimated breast cancer incidence of 20-25 per 100,000 women, second only to cervical cancer [2]. Age-standardized incidence rates are comparatively lower in Senegal (22.4 per 100,000) than other regional rates (i.e. Nigeria 50.4 per 100,000), although limited data and cancer registries may alter the estimates of population incidence. Despite the lower overall incidence of breast cancer, survival rates are significantly lower in Africa when compared to North America; while breast cancer survival rates approach 80% in North America, survival rates are below 40% in low income countries [3]. Socio-economic factors that limit access to health care contribute to this disparity. Genetic factors and breast cancer phenotypes may also influence the aggressive breast cancer presentations and outcomes in African women [4]. Prior studies have found that women in some African countries present later in the disease course, are affected at younger ages, and have more aggressive breast cancer phenotypes including 61% triple negative phenotypes from a study in Ghana [5]. Other studies from the Ivory Coast, Nigeria, and Uganda found lower rates of triple negative breast cancers (30-35%) suggesting genetic and demo-graphic variability [6-9]. In this study, we further characterize the clinic-pathologic characteristics of breast cancers in women presenting with masses in Senegal, West Africa.

## Methods

**Patient selection:** as part of the Early Detection Research Network, 522 Senegalese women presenting consecutively from February 2001 through April 2006 to the Dantec Hospital of the University of Dakar Tumor Institute with masses clinically diagnosed as breast cancer and who had not undergone previous biopsy, surgery, or therapy, were enrolled in the study. Women with a clinical diagnosis of breast cancer were enrolled for pathological evaluation of the suspected breast cancer. After providing informed consent, the study participants were interviewed to elicit information regarding demographic characteristics, gynecologic history, use of cigarettes and alcohol, family history of cancer, and medical history. All women underwent a physical examination and medical history, and in a subset of women, blood was collected for methylation studies as previously described and reported separately [10]. Size of the presenting breast mass was estimated by clinical examination. Information regarding study patient demographic characteristics was collected on site in study questionnaires, entered into an access database, and later linked to pathologic characteristics, which were ascertained on core biopsy samples subsequently reviewed at the University of Washington, in Seattle. Data analyses were performed using STATA version 14.1.414. Venn diagrams were created using BioVenn [11].

**Biopsy and pathology methods:** written informed consent was obtained in compliance with the Human Subjects Institutional Review Boards of the University of Washington (Seattle, WA) and the University of Dakar. A palpation guided needle core biopsy was performed on the presenting masses and fixed in formalin with subsequent histologic examination on H&E stain. While ~200 women evaluated based on palpation guided fine needle aspiration, that information was not available for review or immunohistochemistry. Initial diagnoses were made by local study pathologists and reviewed centrally by a single pathologist (author KHA) for further characterization of histologic subtype, grade (using the Nottingham scoring system). Needle core biopsies with available additional tissue were stained by immunohistochemistry (IHC) for estrogen receptor (ER), progesterone receptor (PR), HER2 and Ki67 and were included in this study [12]. Tissue sections were deparaffinized followed by blockade of endogeneous peroxidases and antigen retrieval using Antigen Unmasking Solution (Vector). ER clone 1D5 using a dilution of 1:1000, following a 15-minute pretreatment in citrate buffer, pH = 6.0 and PR (BioGenex) clone PR88 using a dilution of 1:100 following an 18-minute pretreatment in citrate buffer pH = 6.0. The slides were then counterstained in hematoxylin, dehydrated, and mounted. Positive and negative external controls were performed. HER2 was defined as positive if IHC was 3+ (strong circumferential membranous staining), with adequate internal and external controls. ER and PR scoring was performed using the Allred scoring system with an Allred score ≥ 3 considered a positive result [13]. Anti-HER2 polyclonal antibody (catalog No. A0485, diluted 1:200; DAKO, Carpinteria, CA) staining was performed and scored using a method previously described [14]. Fluorescence in situ hybridization (FISH) for HER2 gene amplification status was performed on HER2 IHC equivocal cases. HER2 FISH was performed manually according to the vendor's protocol using the US Food and Drug Administration-approved Vysis PathVysion (Abbott-Vysis, Des Plaines, IL) using methods previously described [15]. A ratio of > 2.0 was considered as a positive result.

## Results

Of the 522 women enrolled, the presence of invasive cancer was confirmed by cytology and/or core biopsy histology in 397 (76.1%) cases. Non-invasive cancer diagnoses included benign abscesses and lesions, fibroadenomas, phyllodes tumors, papillomas, and ductal carcinoma in situ. Eighteen subjects had insufficient samples for diagnosis. Of confirmed 397 invasive cancer cases, 197 were subsequently stained by IHC for ER, PR and HER2 performed on needle core biopsy specimens. Mean age at presentation was 47.0 years, and 54.4% were premenopausal (Table 1). Mean gravidity was high (5.7 pregnancies/women), and study subjects rarely had a reported history of hormonal contraception (10%), alcohol use (1.0%), or cigarette smoking (0.5%). Only 1.6% reported a positive family history of breast cancer. Most women presented with late stage disease (41.8% stage III and 39.7% stage IV) and tumors were large (mean 7.5 cm). The predominant histologic subtype of cancer was invasive ductal carcinoma (which was considered synonymous with the WHO “no special type” (98%), with the remainder being invasive lobular carcinomas (2%). Using the Nottingham grading system, 3.6% were classified as grade I, 20.2% as grade 2, and 76.2% as grade 3. Overall, 41.1% of invasive breast cancer cases were ER positive, 26.9% were PR positive, and 14.7% were HER2 positive.

**Table 1:** Characteristics of histologically confirmed breast cancer from Senegal (N=197)

Age (mean years ± SD)	47.0 (+/-12.7)
Pre-Menopausal	105 (53.3)
Gravidity (mean ± SD)	5.7 (+/-3.2)
**Ethnicity (N = 195)**	
Wolof	100 (51.3)
Pular	54 (27.7)
Serere	16 (8.2)
Other	25 (12.8)
Known history of hormonal contraception	12 (10.0)
Alcohol Use	2 (1.0)
Cigarette Smoking	1 (0.5)
Family history of breast cancer	3 (1.6)
**Stage**	
I	4 (2.1)
II	32 (16.5)
III	81 (41.8)
IV	77 (39.7)
Tumor Size (mean cm ± SD)	7.5 (4.2)
**Histologic Type**	
Ductal, NOS	192 (98.0)
Lobular	4 (2.0)
**Nottingham Grade**	
1	7 (3.6)
2	39 (20.2)
3	147 (76.2)
Ki-67 Score (mean ± SD)	44. (25.9)
**Tumor Subtypes**	
ER+	81 (41.1)
PR+	53 (26.9)
HER2+	29 (14.7)
ER+/PR+	85 (43.1)
ER-/PR-/HER2-	92 (46.7)

**Figure 1:**
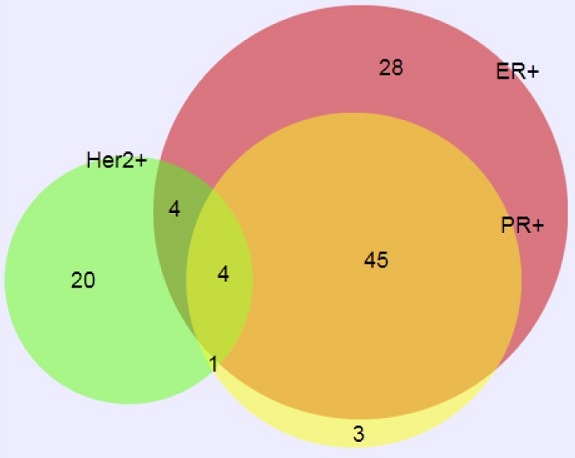
Distribution of ER, PR, and HER2 in non-triple negative breast cancers in Senegal

Nearly half (92/197, 46.7%) were negative for ER, PR and HER2. Among the remaining 105 cases which were positive for ER, PR and/or HER2, most PR positive cases (49/53) were also ER positive (Figure 1). In contrast, most HER2 positive cases (20/29) were ER negative/PR negative. Only 4 of 197 cases were positive for ER, PR, and HER2. The morphology and grade of the invasive cancers was in concordance with the immunohistochemical profile (e.g. cases with basal-like, high grade features were most often triple negative, while well-differentiated, low grade cases were ER+/PR+, etc.), arguing against poor fixation or storage issues. To assess demographic and tumor related factors associated with IHC findings, we classified cases into four clinical treatment IHC groups by combined ER/PR status and HER2 status: ER-/PR-, HER2- (n=92; 46.7%), ER-/PR-, HER2+ (n=20; 10.1%), ER+/PR+, HER2- (n=76; 38.6%) and ER+/PR+, HER2+ (n=9; 4.6%). Age at time of diagnosis was similar between these four IHC groups (Table 2) although more HER2 positive cases were premenopausal (p=0.05). Stage of disease at diagnosis differed by IHC group (p=0.008), with 80% of ER-/PR-, HER2+ cancers presenting at Stage 4 compared to 35.6% of ER-/PR-, HER2- and 32% of ER+/PR+, HER2- cancers. Tumor size was similar across the groups (p=0.5), but ER+/PR+, HER2- cancers were significantly lower Nottingham grade compared to the other three groups (p<0.001). Similarly, more ER+/PR+, HER2- cancers had Ki-67 scores below 20%, compared to other women (p<0.001).

**Table 2:** Characteristics of histologically confirmed invasive breast cancers from Senegal, by ER/PR and HER2 status (n=197)

	ER-/PR -		ER+/PR +		
	HER2-(n=92)	HER2+(n=20)	HER2-(n=76)	HER2+(n=9)	P-value
**Age**					0.3
<40	19 (21.1)	5 (25.0)	21 (28.4)	5 (55.6)	
40-49	29 (32.2)	8 (40.0)	27 (36.5)	1 (11.1)	
50-59	22 (24.4)	2 (10.0)	9 (12.2)	1 (11.1)	
60+	20 (22.2)	5 (25.0)	17 (23.0)	2 (22.2)	
Pre-Menopausal	40 (44.4)	13 (65.0)	45 (60.8)	7 (77.8)	0.05
**Gravidity**					0.10
0	1 (1.1)	1 (5.0)	4 (5.6)	0 (0.0)	
1-3	19 (21.4)	1 (5.0)	20 (27.8)	5 (55.6)	
4-6	32 (36.0)	9 (45.0)	22 (30.6)	3 (33.3)	
7-9	21 (23.6)	7 (35.0)	21 (29.2)	1 (11.1)	
10+	16 (18.0)	2 (10.0)	5 (6.9)	0 (0.0)	
**Ethnicity**					0.24
Wolof	48 (53.3)	9 (45.0)	38 (50.0)	5 (55.6)	
Pular	25 (27.8)	4 (20.0)	21 (27.6)	4 (44.4)	
Serere	6 (6.7)	5 (25.0)	5 (6.6)	0 (0.0)	
Other	11 (12.2)	2 (10.0)	12 (15.8)	0 (0.0)	
**Stage**					0.008
I	4 (4.4)	0 (0.0)	0 (0.0)	0 (0.0)	
II	14 (15.6)	1 (5.0)	15 (20.0)	2 (22.2)	
III	40 (44.4)	3 (15.0)	36 (48.0)	2 (22.2)	
IV	32 (35.6)	16 (80.0)	24 (32.0)	5 (55.6)	
**Tumor Size**					0.5
≤3 cm	10 (11.4)	1 (5.3)	9 (12.5)	1 (11.1)	
4-6 cm	35 (39.8)	9 (47.4)	30 (41.7)	3 (33.3)	
7-9 cm	9 (10.2)	1 (5.3)	13 (18.1)	3 (33.3)	
10+ cm	34 (38.6)	8 (42.1)	20 (27.8)	2 (22.2)	
**Invasion Type**					0.5
Ductal	91 (98.9)	19 (100.0)	73 (96.0)	9 (100.0)	
**Nottingham Grade**					<0.001
1	1 (1.1)	0 (0.0)	6 (8.0)	0 (0.0)	
2	10 (11.2)	0 (0.0)	28 (37.3)	1 (11.1)	
3	78 (87.6)	20 (100.0)	41 (54.7)	8 (88.9)	
**Ki-67 Score**					<0.001
<20	6 (7.8)	0 (0.0)	20 (29.9)	0 (0.0)	
20-50	35 (45.5)	10 (62.5)	26 (38.8)	6 (75.0)	
51-79	15 (19.5)	6 (37.5)	12 (17.9)	2 (25.0)	
80+	21 (27.3)	0 (0.0)	9 (13.4)	0 (0.0)	

## Discussion

Women presenting with a breast mass in Senegal, West Africa, are often clinically diagnosed with breast cancer without biopsy confirmation. A recent meta-analysis estimated the crude incidence of breast cancer in Africa found that of the 2,858 regional breast cancer studies, 782 studies were excluded due to paucity of data on breast cancer incidence or incomplete pathology data and an additional 142 studies were excluded for an unclear description of cancer registration and diagnostic criteria. Of the 2,858 studies, only 41 had sufficient pathology and diagnostic criteria data to include in the analysis [16]. This emphasizes the importance of pathologic diagnosis for clinical breast cancer estimates to provide accurate estimation of breast cancer incidence and subtypes as well as appropriate clinical management. In our study, clinical breast cancer diagnoses were confirmed by biopsy and subsequent immunohistochemical staining. The histologically confirmed breast cancers in this population typically presented in young, highly parous women and were most commonly high grade, invasive ductal carcinoma with a high stage at presentation. Epidemiological data from sub-Saharan Africa suggests that breast cancers occur at a younger age and has more aggressive pathologic features, including high rates of ER-negative, grade 3 tumors presenting at later stages at diagnosis than in populations in the United States and Europe [3, 8, 16-19]. Specifically, in Tanzania 71% of patients with non-metastatic breast cancer were stage 3 at the time of presentation. Similarly, 78% in Mali, 60% in Egypt, and 69% in Uganda presented with stage 3 disease [2, 20, 21]. West Africa is the founder population of most African Americans, and in our study, there were similarities to African American breast cancer phenotypes and presentation. For example, the African American women presented with higher stage disease than non-African American women (51% vs. 39% with Stage II vs. Stage I) [19].

Most strikingly, almost half of Senegalese breast cancers were triple negative, a much higher proportion than observed studies in East and North African countries, such as Uganda, where the rates of triple negative breast cancers are comparatively lower (34%) and more comparable to the United States and Europe [6, 8, 9, 20, 22]. Our data is more similar to other West African studies. For example, a study from Ghana found that 61% of breast cancers were triple negative [6]. This high rate of triple negative breast cancers in West Africa, the founder population of most African Americans, is in keeping with the clinico-pathologic features of breast cancers among non-Hispanic black women in the United States (younger age at presentation and higher frequency of triple negative cancers than the general population) [19]. These findings suggest a genetic and/or epigenetic influence, which has recently been discovered with data from the Cancer Genome Atlas. The CGA analysis found higher frequencies of estrogen receptor-negative breast cancer risk alleles in blacks than whites in the United States, providing further molecular basis of ER-negative phenotypes [23]. Additional studies exploring the genetic and epigenetic basis of these aggressive phenotypes are warranted. HER2+ breast cancers represented 14.7% of the breast cancers in our study, and were more commonly stage IV at diagnosis. This aggressive behavior is in keeping with the known aggressive clinical course in the era before available targeted therapy [24]. Given the high percentage of late stage diagnoses in our Senegalese cohort, we might have expected a much higher HER2+ percentage than 14.7%. This inconsistency raises the possibility of additional genetic and/or epigenetic influences specific to the Senegelese population.

While predisposing genetic factors seem to explain some of the differences, geneenvironment interactions are also implicated in the development of aggressive breast cancer phenotypes [25]. Specifically, transcriptional silencing of tumor suppressor genes via methylation of CpG islands in promoter regions is thought to be an early step in carcinogenesis, and is largely influenced by environmental exposures such as biopsychosocial stressors [25]. Our previously published methylation data performed in this same cohort of women analyzed 32 genes known to be involved in hormone signaling, cellular proliferation, and DNA repair in breast carcinoma [10]. Five of these genes were found to be significantly methylated in breast cancers from our cohort in Senegal (APC, RASSF1, GASTP1, SCGB3a1, and HS3ST2). Hypermethylation was found more often in ER positive/PR positive and HER2 positive cancer whereas hypomethylation was more common in triple negative cancers, which is similar to other such methylation studies [10]. These epigenetic changes point to a complex interaction of genes and environment in the development of the aggressive phenotypes of cancers found in our study.

## Conclusion

Our study contributes to the knowledge of the frequency of breast cancers with histologic confirmation. We found that breast cancers from Senegal tend to have aggressive clinico-pathologic features. While it is still unclear what the relative contributions of population-specific differences account for the distinctive breast cancer phenotypes in Senegal (such as mean age at presentation, screening programs and genetic/regional risk factors), a better understanding of the frequencies of these breast cancers phenotypes will help guide diagnosis and treatment.

### What is known about this topic

- Breast cancers in sub-Saharan African women occur at a younger age with more aggressive pathologic features;
- Breast cancers from some countries in West Africa have high rates of ER-negative, grade 3 tumors that present at later stages at diagnosis than in populations in the United States and Europe;
- Hypermethylation of five genes in our study was found more often in ER positive/PR positive and HER2 positive cancer whereas hypomethylation was more common in triple negative cancers.

### What this study adds

- Information about histologically confirmed breast cancers in an understudied region of Africa;
- Breast cancers from Senegal tend to have aggressive clinico-pathologic features;
- The inconsistency between late stage of presentation and relatively modest rates of HER2+ breast cancers raises the possibility of additional genetic or epigenetic influences in this population.

## Competing interests

The authors declare no competing interests.

## References

[1] International Agency for research on cancer World Health Organization. Global cancer observatory.

[2] Ly M, Valent A, Diallo G, Penault-Lorca F, Dumke K, Marty V (2013). Gene copy number variations in breast cancer of Sub-Saharan African women. The Breast.

[3] Coleman MP, Quaresma M, Berrino F, Lutz JM, De Angelis R, Capocaccia R (2008). Cancer survival in five continents: a worldwide population-based study (CONCORD). Lancet Oncol.

[4] Huo D, Ikpatt F, Khramtsov A, Dangou JM, Nanda R, Dignam J (2009). Population Differences in Breast Cancer: Survey in Indigenous African Women Reveals Over-Representation of Triple-Negative Breast Cancer. J Clin Oncol.

[5] Dem A, Traoré B, Dieng MM, Diop PS, Ouajdi T, Lalami MT (2008). Gynaecological and breast cancers at the Dakar Cancer Institute. Sante.

[6] Proctor E, Kidwell KM, Jiagge E, Bensenhaver J, Awuah B, Gyan K (2015). Characterizing breast cancer in a population with increased prevalence of triple-negative breast cancer: androgen receptor and ALDH1 expression in Ghanaian Women. Ann Surg Oncol.

[7] Gueye M, Gueye SMK, Diallo M, Thiam O, Mbodji A, Diouf A (2017). Sociodemographic factors associated with delays in breast cancer. Open J Obstet Gynecol.

[8] Miguel F, Lopes LV, Ferreira E, Ribas E, Pelaez AF, Leal C (2017). Breast cancer in Angola, molecular subtypes: a first glance. Ecancermedicalscience.

[9] Effi AB, Aman NA, Koui BS, Koffi KD, Traore ZC, Kouyate M (2016). Breast Cancer Molecular Subtypes Defined by ER/PR and HER2 Status: association with Clinicopathologic Parameters in Ivorian Patients. Asian Pac J Cancer Prev.

[10] Hoque MO, Feng Q, Toure P, Dem A, Critchlow CW, Hawes SE (2006). Detection of Aberrant Methylation of Four Genes in Plasma DNA for the Detection of Breast Cancer. J Clin Oncol.

[11] Hulsen T, de Vlieg J, Alkema W (2008). BioVenn: a web application for the comparison and visualization of biological lists using area-proportional Venn diagrams. BMC Genomics.

[12] Elston CW, Ellis IO (2002). Pathological prognostic factors in breast cancer. I. The value of histological grade in breast cancer: experience from a large study with long-term follow-up. Histopathology.

[13] Fitzgibbons PL, Murphy DA, Hammond MEH, Allred DC, Valenstein PN (2010). Recommendations for validating estrogen and progesterone receptor immunohistochemistry assays. Arch Pathol Lab Med.

[14] Grimm EE, Schmidt RA, Swanson PE, Dintzis SM, Allison KH (2010). Achieving 95% Cross-Methodological Concordance in HER2 Testing: Causes and Implications of Discordant Cases. Am J Clin Pathol.

[15] Allison KH, Dintzis SM, Schmidt RA (2011). Frequency of HER2 heterogeneity by fluorescence in situ hybridization according to CAP expert panel recommendations: time for a new look at how to report heterogeneity. Am J Clin Pathol.

[16] Adeloye D, Sowunmi OY, Jacobs W, David RA, Adeosun AA, Amuta AO (2018). Estimating the incidence of breast cancer in Africa: a systematic review and meta-analysis. J Glob Health.

[17] Silverstein A, Sood R, Costas-Chavarri A (2016). Breast Cancer in Africa: Limitations and Opportunities for Application of Genomic Medicine. Int J Breast Cancer.

[18] Bray F, Ren J-S, Masuyer E, Ferlay J (2013). Global estimates of cancer prevalence for 27 sites in the adult population in 2008. Int J Cancer.

[19] Carey LA, Perou CM, Livasy CA, Dressler LG, Cowan D, Conway K (2006). Race, Breast Cancer Subtypes, and Survival in the Carolina Breast Cancer Study. JAMA.

[20] Jin Z, Tamura G, Tsuchiya T, Sakata K, Kashiwaba M, Osakabe M (2001). Adenomatous polyposis coli (APC) gene promoter hypermethylation in primary breast cancers. Br J Cancer.

[21] Galukande M, Wabinga H, Mirembe F, Karamagi C, Asea A (2014). Molecular breast cancer subtypes prevalence in an indigenous Sub Saharan African population. Pan Afr Med J.

[22] Popoola AO, Ogunleye OO, Ibrahim NA, Omodele FO, Igwilo AI (2012). Five year survival of patients with breast cancer at the Lagos State University Teaching Hospital, Nigeria. Online J Med Med Sci Res.

[23] Huo D, Hu H, Rhie SK, Gamazon ER, Cherniack AD, Liu J (2017). Comparison of Breast Cancer Molecular Features and Survival by African and European Ancestry in The Cancer Genome Atlas. JA-MA Oncol.

[24] Slamon DJ, Clark GM, Wong SG, Levin WJ, Ullrich A, McGuire WL (1987). Human breast cancer: correlation of relapse and survival with amplification of the HER-2/neu oncogene. Science.

[25] Linnenbringer E, Gehlert S, Geronimus AT (2017). Black-white disparities in breast cancer subtype: the intersection of socially patterned stress and genetic expression. AIMS Public Health.

